# Synthesis and *In-vitro* Cytotoxicity Assessment of *N-*(5-(Benzylthio)-1,3,4- thiadiazol-2-yl)-2-(4-(trifluoromethyl)phenyl)acetamide with Potential Anticancer Activity 

**Published:** 2013

**Authors:** Alireza Aliabadi, Zaman Hasanvand, Amir Kiani, Seyed Saber Mirabdali

**Affiliations:** a Department of Medicinal Chemistry, Faculty of Pharmacy, Kermanshah University of Medical Sciences, Kermanshah, Iran.; b Student Research Committee, Kermanshah University of Medical Sciences, Kermanshah, Iran.; c Department of Pharmacology, Toxicology and Medical Services, Faculty of Pharmacy, Kermanshah University of Medical Sciences, Kermanshah, Iran.

**Keywords:** Synthesis, 1,3,4-Thiadiazole, MTT assay, Breast cancer

## Abstract

Cancer is the second leading cause of death in the world. Despite advances in the diagnosis and treatment, overall survival of patients still remains poor. Hence, there is an urgent need for development of new anticancer agents. Considering promising biological activity of 1,3,4-thiadiazole derivatives, in the present study, synthesis and cytotoxicity assessment of new derivatives of this ring was done. All synthesized compounds were characterized by NMR, IR and MS spectroscopic methods. Obtained data from MTT assay showed that all compounds 3a- 3l had better anticancer activity against MDA(breast cancer) compared to PC3(prostate cancer) and U87(Glioblastoma). Compound 3 g with m-OCH3 moiety on the phenyl ring was the most potent one in this series with IC_50_ = 9 μM against MDA breast cell line in comparison with imatinib (IC_50_ = 20 μM) as reference drug.

## Introduction

Cancer is a disease in which cells can be aggressive, invasive and/or metastatic. These are three malignant properties of cancer cells that differentiate them from benign tumors which are self-limited in their growth and do not invade or metastasize (1-4). Cancer is the second leading cause of death in the world. Despite advances in the diagnosis and treatment, overall survival of patients still remains poor. Until recently, surgery, chemotherapy, radiotherapy and endocrine therapyhave been the standard treatment options available for cancer patients. This has improved survival in several types of solid tumors; however, drug toxicity and emergence of drug resistance have been the major causes of failure in treatment. Hence, there is an urgent need for discovery of new anticancer agents to overcome the disadvantages of the currently available anticancer drugs (3, 5-7). 

**Figure 1:**
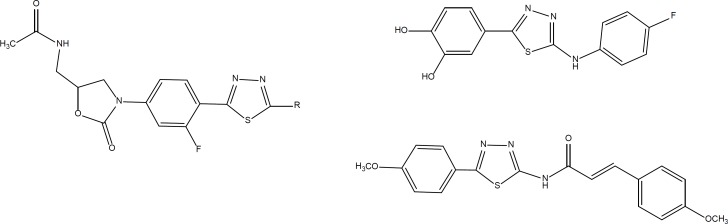
Structures of some 1,3,4-thiadiazole-based compounds with anticancer activity

**Figure 2:**
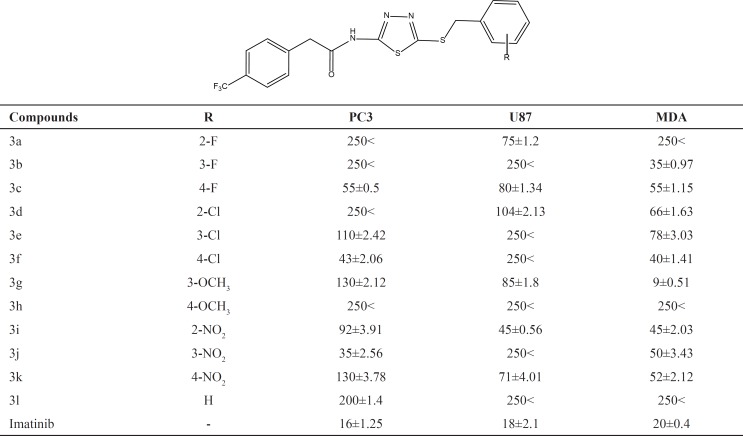
Total structure of 5-amino-1,3,4-thiadiazole-2-thiol derivatives as anticancer agents

Diverse chemical structure containing 1,3,4-Thiadiazole have been reported with potential anticancer activity (Figure 1) (8-13). Recently Maurizio Botta and coworkers reported the discovery of new derivatives of *N*-(5- (benzylthio)-1,3,4-thiadiazol-2-yl) benzamide as potent dual inhibitors of abl and src tyrosine kinases (14, 15) (Figure 2). In the present study, we synthesized a new series of these derivatives and evaluated their preliminary anticancer activity *in-vitro *against three cancer cell lines. 

## Results and Discussion

According to Table 1, all synthesized compounds 3a-3l were evaluated against three cancer cell lines. Overall, the best anticancer activity were observed against MDA breast cancer cell line compared to other cell lines (PC3 and U87). Substitution of various moieties such as F, Cl, nitro and methoxy were done at different positions of the phenyl ring to study the electronic effects of the related substituents. 

**Table 1:** Cytotoxicity results (IC_50_, μM) of compounds 3a-3l against cancerous cell lines, PC3 (Prostate cancer), U87(Glioblastoma) and MDA(Breast cancer).

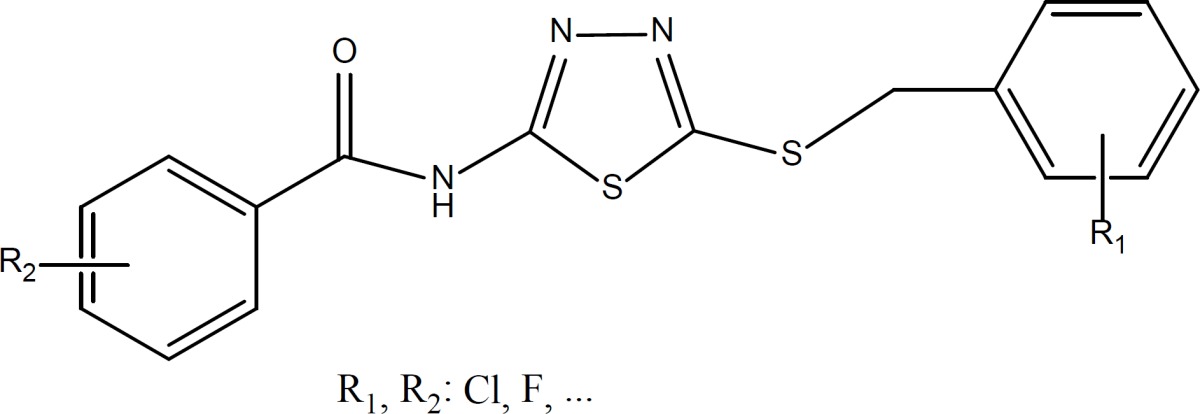

Fluorine substitution showed the best anticancer properties against MDA cell line at position 3(*meta*) of the phenyl ring (compound 3b) with IC_50_ = 35 μM. Comparison of cytotoxic effects of fluorinated derivatives in all cell lines exhibited that *para *position is the best position for rendering of the optimal activity(compound 3c). Chlorinated derivatives demonstrated better cytotoxic activity at position *para *(compound 3f) compared to other positions of the phenyl ring. Compound 3f rendered more cytotoxic activity toward PC3 and MDA cell lines. Investigation of the role of methoxy group at positions *meta *and *para *caused a high cytotoxic effects for compound 3g with *m*-methoxy substituent in all cell lines. This compound showed higher activity with IC_50_ = 9 μM compared to imatinib (IC_50 _= 20 μM) in MDA breast cancer cell line. Substitution of nitro group at position 2 of the phenyl ring led to an acceptable cytotoxicity against U87 and MDA cell lines rather than PC3 cell line. Nitro moiety rendered the best anticancer activity at position 3(*meta*) against PC3 cell line. Insertion of the phenyl ring without any moiety resulted in implausible effects in all cell lines. 

## Experimental

*Chemistry*

All chemical compounds were purchased from commercial suppliers of Merck and Aldrich companies. The purity of the prepared compounds was proved by thin layer chromatography (TLC) using various solvents of different polarities. Merck silica gel 60 F_254_ plates were applied for analytical TLC. Column chromatography was performed on Merck silica gel (70-230 mesh) for purification obtained compounds. 1H-NMR spectra were recorded using a Brucker 200 MHz spectrometer, and chemical shifts were expressed as *δ *(ppm) with tetramethylsilane (TMS) as internal standard. The IR spectra were obtained on a Shimadzu 470 spectrophotometer (potassium bromide disks). Melting points were determined using electrothermal melting point analyzer apparatus and were uncorrected. The mass spectra were run on a Finigan TSQ-70 spectrometer (Finigan, USA) at 70 eV. All cell lines were purchased from the Pasteur Institute of Iran. 

**Figure 3:**
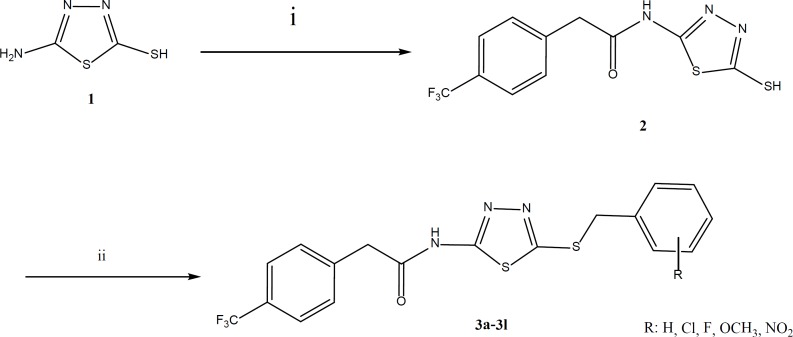
Synthetic procedure of compounds 3a-3l, Reagents and conditions i) 4-Trifluoromethylphenylacetic acid, EDC, HOBt, CH3CN, rt, 24 h, ii) Benzyl chloride derivatives, KOH, EtOH, reflux, 24 h

According to the Figure 3, 5-amino- 1,3,4-thiadiazole-2-thiol (1) was treated with 4-trifluoromethylphenylacetic acid for direct coupling of acid with amine. The reaction was carried out in the presence of EDC and hydroxybenzotriazole (HOBt) in acetonitrile as solvent. The termination of reaction was proved by thin layer chromatography (TLC). After completion, the solvent was evaporated using rotary evaporator apparatus and ethyl acetate and water were added. The aqueous phase was removed and the organic phase was washed two times by sodium bicarbonate 5%, diluted sulfuric acid and brine (16-19). Anhydrous sodium sulfate was added for drying and filtration was done. Ethyl acetate was removed under reduced pressure and a yellow powder was obtained. The obtained product was used after crystallization from ethanol for the next step. Various benzyl chloride derivatives were reacted with compound 2 for obtaining the final appropriate products (3a-3l). The 1H NMR, IR and MS spectra were used to confirm the synthesized compounds.

*Synthesis of N-(5-Mercapto-1,3,4-thiadiazol-2-yl)-2-(4-(trifluoromethyl)phenyl)acetamide (2)*

In a flask, equimolar amounts of 4-trifluoromethylphenylacetic acid, EDC and HOBtin acetonitrile solvent were stirred for 30 min and then equimolar quantity of 5-amino-1,3,4-thiadiazole-2-thiol was added. The stirring condition was continued for 24 h. The end point of the reaction was determined by thin layer chromatography(TLC). Acetonitrile was removed under reduced pressure and ethyl acetate/water was added. The aqueous layer was removed and organic layer was washed two times by sodium bicarbonate 5%, diluted sulfuric acid and brine. Anhydrous sodium sulfate was added for drying and then filtered. The ethyl acetate was evaporated using rotary evaporator apparatus. The obtained yellowish solid was washed by dry ether and used for the next step.

mp. 171°C, Yield: 65%,C_11_H_8_F_3_N_3_OS_2_, MW: 319 g/mol,1H NMR (DMSO-d6, 200 MHz) *δ*: 3.76 (s, 2H, -CH_2_CO-), 3.95 (s, 1H, -SH), 7.58 (m, 2H, *J *= 8Hz), 8.19 (m, 2H, *J *= 8Hz, 4-trifluoromethylphenyl), 12.76 (brs, 1H, NH).IR (KBr, cm-1) ῡ: 3265, 1697, 1580, 1519, 1321, 1155, 1103, 1064, 821, 705.MS(m/z, %): M^++2^: 322(10), M^+^: 320(10), 279(45), 276(35), 167(80), 159(95), 149(100), 133(15), 109(15), 71(15), 57(20).

*General procedure for synthesis of compounds 3a-3l*

Equimolar quantities of appropriate benzyl chloride derivative was treated with 2-(4-fluorophenyl)-*N*-(5-mercapto-1,3,4-thiadiazol-2-yl)acetamide (2). Equimolar amount of potassium hydroxide in absolute ethanol was added to convert the thiol moiety to the thiolate anion. Then, the related benzyl chloride derivative was added to the reaction medium and reflux condition was performed for 24 h. Crushed ice was added and the precipitate filtered, washed by cool water and purified by appropriate procedures such as crystallization or column chromatography (EtOAC/Petroleum ether: 3/2). 

*N-(5-(2-Fluorobenzylthio)-1,3,4-thiadiazol-2-yl)-2-(4-(trifluoromethyl)phenyl)acetamide (3a)*

mp. 201 °C, Yield: 42%,C_18_H_13_F_4_N_3_OS_2_, MW: 427 g/mol,1H NMR (DMSO-d_6_, 200 MHz) *δ*: 4.00 (s, 2H, -CH_2_CO-), 4.53 (s, 2H, -CH_2_S-), 7.16-7.54 (m, 2-fluorobenzyl), 7.59 (d, 2H, *J *= 8 Hz, 4-trifluoromethylphenyl), 7.76 (d, 2H, *J *= 8 Hz, 4-trifluoromethylphenyl), 13.00 (s, NH). IR(KBr, cm^-1^) ῡ: 3158, 3050, 2898, 1690, 1560, 1493, 1456, 1421, 1402, 1337, 1301, 1236, 1168, 1117, 1071, 1019, 972, 844, 757, 693, 655.MS(m/z, %): M^+^: 427(95), 377(60), 325(35), 297(30), 236(25), 236(15), 159(75), 109(100). 

*N-(5-(3-Fluorobenzylthio)-1,3,4-thiadiazol-2-yl)-2-(4-(trifluoromethyl)phenyl)acetamide (3b)*

mp. 190 °C, Yield: 58%,C_18_H_13_F_4_N_3_OS_2_, MW: 427 g/mol,1H NMR (DMSO-d_6_, 200 MHz) δ: 3.93 (s, 2H, -CH_2_CO-), 4.53 (s, 2H, -CH_2_S-), 7.01-7.47 (m, 3-fluorobenzyl), 7.58 (d, 2H, *J *= 8 Hz, 4-trifluoromethylphenyl), 7.75 (d, 2H, *J *= 8 Hz, 4-trifluoromethylphenyl), 12.98 (s, NH).IR(KBr, cm^-1^) ῡ: 3448, 3156, 2916, 1699, 1562, 1488, 1358, 1326, 1172, 1110, 1068, 837.MS(m/z, %): M^+^: 427(85), 377(80), 325(40), 297(25), 236(20), 159(70), 109(100). 

*N-(5-(4-Fluorobenzylthio)-1,3,4-thiadiazol-2-yl)-2-(4-(trifluoromethyl)phenyl)acetamide (3c)*

mp. 166 °C, Yield: 40%,C_18_H_13_F_4_N_3_OS_2_, MW: 427 g/mol,1H NMR (DMSO-d_6_ , 200 MHz) *δ*: 3.99 (s, 2H, -CH_2_CO-), 4.51 (s, 2H, -S-CH_2_-), 7.24 (t, 2H, 4-fluorobenzyl), 7.48 (t, 2H, 4-fluorobenzyl), 7.58 (d, 2H, *J *= 8 Hz, 4-trifluoromethylphenyl), 7.75 (d, 2H, *J *= 8 Hz, 4-trifluoromethylphenyl), 12.97 (s, NH).IR(KBr, cm-1) ῡ: 3370, 3030, 2920, 2829, 1701, 1558, 1508, 1323, 1219, 1160, 1109, 1060, 835.MS(m/z, %): M+: 427(90), 377(75), 325(40), 297(30), 236(25), 159(75), 109(100). 

*N-(5-(2-Chlorobenzylthio)-1,3,4-thiadiazol-2-yl)-2-(4-(trifluoromethyl)phenyl)acetamide (3d)*

mp. 208 °C, Yield: 54%,C_18_H_13_ClF_3_N_3_OS_2_, MW: 443 g/mol,1H NMR (DMSO-d6, 200 MHz) *δ*: 4.00 (s, 2H, -CH_2_CO-), 4.59 (s, 2H, -CH2S-), 7.35 (m, 2-chlorophenyl), 7.48 (d, 2H, *J *= 12 Hz, 4-trifluoromethylphenyl), 7.55 (m, 2-chlorophenyl), 7.97(d, 2H, *J *= 12 Hz, 4-trifluoromethylphenyl), 13.00 (s, NH). IR(KBr, cm^-1^) ῡ: 3447, 3165, 2922, 1700, 1570, 1445, 1326, 1171, 1109, 1067, 868, 759.MS(*m/z*, %): M^+^:443(10), 410(55), 409(85), 159(40), 127(50), 125(100), 109(10), 89(10). 

*N-(5-(3-Chlorobenzylthio)-1,3,4-thiadiazol-2-yl)-2-(4-(trifluoromethyl)phenyl)acetamide (3e)*

mp. 198 °C, Yield: 57%,C_18_H_13_ClF_3_N_3_OS_2_, MW: 443 g/mol,1H NMR (DMSO-d6 , 200 MHz) *δ*: 4.00 (s, 2H, -CH_2_CO-), 4.59 (s, 2H, -S-CH2-), 7.33-7.40 (m, 3-chlorobenzyl), 7.43 (d, 2H, *J *= 8 Hz, 4-trifluoromethylphenyl), 7.56 (m, 3-chlorobenzyl), 7.75 (d, 2H, *J *= 8 Hz, 4-trifluoromethylphenyl), 13.00 (s, NH).IR(KBr, cm^-1^) ῡ: 3427, 3165, 2914, 2730, 1700, 1569, 1446, 1356, 1326, 1301, 1170, 1112, 1066, 868, 839, 759.MS(*m/z*, %): M^+^:443(15), 410(60), 409(65), 282(10), 159(55), 127(35), 125(100), 89(10).

*N-(5-(4-Chlorobenzylthio)-1,3,4-thiadiazol-2-yl)-2-(4-(trifluoromethyl)phenyl)acetamide (3f)*

mp. 198 °C, Yield: 34%,C_18_H_13_ClF_3_N_3_OS_2_, MW: 443 g/mol,1H NMR (DMSO-d_6_, 200 MHz) *δ*: 3.99 (s, 2H, -CH_2_CO-), 4.52 (s, 2H, -S-CH_2_-), 7.38-7.48 (m, 4-chlorobenzyl), 7.58 (d, 2H, *J *= 8 Hz, 4-trifluoromethylphenyl), 7.78 (d, 2H, *J *= 8 Hz, 4-trifluoromethylphenyl), 12.97 (s, NH).IR(KBr, cm^-1^) ῡ: 3440, 3130, 3040, 2877, 1690, 1556, 1334, 1163, 1118, 1068, 1020, 846, 745, 702.MS(*m/z*, %): M++1: 444(10), M^+^:443(10), 410(60), 409(80), 282(12), 178(12), 159(40), 127(50), 125(100), 109(10), 89(10).

*N-(5-(3-Methoxybenzylthio)-1,3,4-thiadiazol-2-yl)-2-(4-(trifluoromethyl)phenyl)acetamide (3g)*

mp. 160 °C, Yield: 32%,C19H16F3N3O2S2, MW: 439 g/mol,1H NMR (DMSO-d_6_ , 200 MHz) *δ*:1H NMR (DMSO-d_6_ , 200 MHz) δ: 3.80 (s, 3H, -OCH_3_), 3.99 (s, 2H, -CH_2_CO-), 4.48 (s, 2H, -S-CH_2_-),7.06(m, 3H, 3-methoxybenzyl), 7.32(m, 1H, 3-methoxybenzyl), 7.58(d, 2H, *J *= 8 Hz, 4-trifluoromethylphenyl), 7.75 (d, 2H, *J *= 8 Hz, 4-trifluoromethylphenyl), 12.97 (s, NH).IR(KBr, cm-1) ῡ:3167, 3038,2946, 1735, 1702, 1607, 1577, 1488, 1438, 1358, 1325, 1302, 1271, 1158, 1113, 1068, 839, 777, 736.MS(*m/z*, %): M^+^: 439(40), 159(75), 122(45), 121(100), 109(35).

*N-(5-(4-Methoxybenzylthio)-1,3,4-thiadiazol-2-yl)-2-(4-(trifluoromethyl)phenyl)acetamide (3h)*

mp. 219 °C, Yield: 36%,C_19_H_16_F_3_N_3_O_2_S_2_, MW: 439 g/mol,1H NMR (DMSO-d6 , 200 MHz) *δ*: 3.76 (s, 3H, -OCH3), 3.99 (s, 2H, -CH_2_CO-), 4.46 (s, 2H, -S-CH_2_-), 6.92 (d, 2H, *J *= 8 Hz, 4-methoxybenzyl), 7.35 (d, 2H, *J *= 8 Hz, 4-methoxybenzyl), 7.58 (d, 2H, *J *= 8 Hz, 4-trifluoromethylphenyl), 7.75 (d, 2H, *J *= 8 Hz, 4-trifluoromethylphenyl), 12.96 (s, NH).IR(KBr, cm^-1^) ῡ: 3265, 3045, 2870, 1691, 1554, 1510, 1400, 1332, 1298, 1170, 1122, 1107, 1066, 827. MS(*m/z*, %): M^++2^: 441(15), M^++1^: 440(20), M^+^: 439(25), 159(60), 122(45), 121(100), 109(30). 

*N-(5-(2-Nitrobenzylthio)-1,3,4-thiadiazol-2-yl)-2-(4-(trifluoromethyl)phenyl)acetamide (3i)*

mp. 146 °C, Yield: 35%,C_18_H_13_F_3_N_4_O_3_S_2_, MW: 454 g/mol,1H NMR (DMSO-d_6_, 200 MHz) *δ*: 4.00 (s, 2H, -CH_2_CO^-^), 4.79 (s, 2H, -CH_2_S-), 7.49 (m, aromatic), 8.11 (m, aromatic), 8.21 (m, aromatic), 13.00 (s, NH). IR(KBr, cm^-1^) ῡ: 3447, 3165, 2923, 1698, 1612, 1573, 1527, 1443, 1335, 1168, 1106, 1066, 830, 827, 702.MS(*m/z*,%): M^+^: 454(15), 270(35), 242(65), 225(15), 195(100), 179(65), 165(85), 136(70), 106(45), 90(50), 78(35).

*N-(5-(3-Nitrobenzylthio)-1,3,4-thiadiazol-2-yl)-2-(4-(trifluoromethyl)phenyl)acetamide (3j)*

mp. 132 °C, Yield: 45%,C_18_H_13_F_3_N_4_O_3_S_2_, MW: 454 g/mol,1H NMR (DMSO-d_6_, 200 MHz) *δ*: 3.84 (s, 2H, -CH_2_CO-), 4.51 (s, 2H, -CH_2_S-), 7.71(m, 5H, aromatic), 8.16(m, 3H, aromatic), 13(brs, NH).IR(KBr, cm-1) ῡ: 3318, 3154, 2850, 1692, 1629, 1562, 1527, 1508, 1487, 1348, 1329, 1163, 1117, 1073, 810, 747.MS(*m/z*,%): M^++1^: 455(7), M^+^: 454(15), 270(30), 242(75), 195(100), 179(70), 165(90), 136(60), 106(35), 90(45), 78(60).

*N-(5-(4-Nitrobenzylthio)-1,3,4-thiadiazol-2-yl)-2-(4-(trifluoromethyl)phenyl)acetamide (3k)*

mp. 198 °C, Yield: 54%,C_18_H_13_F_3_N_4_O_3_S_2_, MW: 454 g/mol,1H NMR (DMSO-d_6_, 200 MHz) *δ*: 3.99 (s, 2H, -CH_2_CO-), 4.66 (s, 2H, -S-CH_2_-),7.55(m, 2H, aromatic), 7.77(m, 4H, aromatic), 8.25(m, 2H, aromatic), 12.99(brs, NH).IR(KBr, cm-1) ῡ:3265, 3153, 3040, 2912, 2852, 1697, 1554, 1517, 1342, 1323, 1155, 1103, 1064, 1020, 960, 830.MS(*m/z*,%): M^++1^: 455(10), M^+^: 454(10), 270(40), 242(75), 225(40), 195(100), 179(95), 165(85), 136(60), 106(50), 90(50), 78(60). 

*N-(5-(Benzylthio)-1,3,4-thiadiazol-2-yl)-2-(4-(trifluoromethyl)phenyl)acetamide (3l)*

mp. 203 °C, Yield: 47%,C_18_H_14_F_3_N_3_OS_2_, MW: 409 g/mol,1H NMR (DMSO-d_6_ , 200 MHz) *δ*: 3.99 (s, 2H, -CH_2_CO-), 4.52 (s, 2H, -S-CH_2_-), 7.30-7.49 (m, 5H, benzyl), 7.58 (d, 2H, *J *= 8 Hz, 4-trifluoromethylphenyl), 7.75 (d, 2H, *J *= 8 Hz, 4-trifluoromethylphenyl), 12.97 (s, NH).IR(KBr, cm^-^1) ῡ: 3375, 3040, 1701, 1556, 1508, 1323, 1294, 1220, 1159, 1107, 1066, 1020, 827, 700.MS(*m/z*, %): M^+^: 410(100), 409(95), 408(95), 159(75), 148(60), 91(75).

*MTT assay*

Diverse derivatives of 1,3,4-thiadiazole (compounds 3a-3l) were tested for cytotoxic activity at 0.1-250 μg/mL concentration in three human cancer cell lines of PC3 cell (prostate cancer), U87 (gliobalstoma) and MDA (breast cancer). Cells from different cell lines were seeded in 96-well plates at the density of 8000–10,000 viable cells per well and incubated for 48 h to allow cell attachment.The cells were then incubated for another 48-96 h (depends to cell cycle of each cell line) with various concentrations of compounds 3a-3l. Cells were then washed inPBS, and 20 μL of MTT (3-(4, 5-dimethylthiazol-2-yl)-2,5-diphenyl tetrazolium bromide solution (5 mg/mL) were added to each well. An additional 4 h of incubation at 37°C were done, and then the medium was discarded. Dimethyl sulfoxide (60 μL) was added to each well, and the solution was vigorously mixed to dissolve the purple tetrazolium crystals. The absorbance of each well was measured by plate reader (Anthous 2020; Austria) at a test wavelength of 550 nm against a standard reference solution at 690 nm. The amount of produced purple formazan is proportional to the number of viable cells (16).
